# Anticoagulation Quality of Warfarin and the Role of Physician–Pharmacist Collaborative Clinics in the Treatment of Patients Receiving Warfarin: A Retrospective, Observational, Single-Center Study

**DOI:** 10.3389/fphar.2020.605353

**Published:** 2021-01-14

**Authors:** Sha Qiu, Na Wang, Chi Zhang, Zhi-Chun Gu, Yan Qian

**Affiliations:** ^1^ Department of Pharmacy, The Second Affiliated Hospital of Chongqing Medical University, Chongqing, China; ^2^ Department of Pharmacy, Renji Hospital, School of Medicine, Shanghai Jiaotong University, Shanghai, China

**Keywords:** warfarin, anticoagulation quality, time in therapeutic range, risk factors, clinical pharmacist

## Abstract

**Background:** The management of patients receiving warfarin is complicated. This study evaluated the anticoagulation quality of warfarin, explored potential predictors associated with poor anticoagulation quality, and elucidated the role of clinical pharmacists in the management of warfarin treatment.

**Methods:** We retrospectively collected data on patients who either initially received warfarin or returned to warfarin after withdrawal between January 1, 2015 and January 1, 2020. The primary outcome was time in therapeutic range (TTR), and a TTR of ≥60% was considered as good anticoagulation quality. The secondary outcomes included thromboembolic and bleeding events during the follow-up. We assessed the TTR of each participant and investigated the potential predictors of poor anticoagulation quality (TTR < 60%) using logistic regression analysis. Additionally, we compared the warfarin anticoagulant quality and the incidence of clinical adverse events between atrial fibrillation patients in physician–pharmacist collaborative clinics (PPCCs) and general clinics.

**Results:** Totally, 378 patients were included. The mean TTR of patients was 42.6 ± 29.8%, with only 32% of patients having achieved good anticoagulation quality. During a mean follow-up period of 192 ± 92 days, we found no significant differences in the incidences of thromboembolic events (5.0% vs. 5.1%, *p* = 0.967) and bleeding events (1.7% vs. 4.7%, *p* = 0.241) between patients with good and those with poor anticoagulation quality. The presence of PPCCs (odds ratio [OR]: 0.47, 95% confidence interval [CI]: 0.25–0.90, *p* = 0.022) was an independent protective factor of poor anticoagulation quality, while the presence of more than four comorbidities (OR: 1.98, 95% CI: 1.22–3.24, *p* = 0.006) and an average interval of international normalized ratio monitoring of >30 days (OR: 1.74, 95% CI: 1.10–2.76, *p* = 0.019) were independent risk factors of poor anticoagulation quality. Compared with atrial fibrillation patients in general clinics, patients in PPCCs were found to have a significantly increased mean TTR level (48.4% ± 25.7% vs. 38.0% ± 27.6%, *p* = 0.014).

**Conclusion:** The anticoagulation quality of warfarin was relatively low at our institution. The presence of more than four comorbidities and an average interval of international normalized ratio monitoring of >30 days independently contributed to poor anticoagulation quality. Meanwhile, the use of PPCC model improved the anticoagulation quality of warfarin.

## Introduction

Although non–vitamin K oral anticoagulants (NOACs), such as dabigatran and rivaroxaban, have been successfully marketed in recent years, warfarin is still widely used in the prevention and treatment of various thromboembolic diseases due to its efficacy and low cost (Gu et al., 2019b). Maintaining the international normalized ratio (INR) within the therapeutic range could potentially optimize the benefit–risk ratio of warfarin treatment (Gu et al., 2019a). Time in therapeutic range (TTR) is commonly applied as a measure of the anticoagulation quality of warfarin therapy within a given time frame (Schmitt et al., 2003). In order to ensure the effectiveness and safety of warfarin in clinical practice, it is necessary to frequently monitor INR and adjust the dosage accordingly. Warfarin has a narrow therapeutic window, and its anticoagulant effect is susceptible to numerous factors, such as diet, drugs, and gene polymorphisms (Gu et al., 2018); hence, tailoring warfarin treatment to the case at hand is a challenge for both patients and physicians. Clinical pharmacists are the main providers of professional pharmaceutical care and can therefore provide good anticoagulant pharmaceutical services to patients and physicians, assisting in the formulation of medication regimens and providing medication education (Zhai et al., 2019). Previous studies have confirmed the important role of clinical pharmacists in improving adherence to anticoagulation treatment (Choe et al., 2002; Garwood et al., 2008; Gupta et al., 2015). However, few studies have focused on the anticoagulation quality of warfarin and the influence of clinical pharmacists in the treatment process. This study aimed to evaluate anticoagulation quality in patients undergoing warfarin treatment at our single center, explore potential predictors associated with poor anticoagulation quality, and elucidate the role of clinical pharmacists in the management of warfarin treatment.

## Methods

### Study Population

This was a retrospective, observational, single-center study in which we analyzed a database of patients who received warfarin at our institution from January 1, 2015 to January 1, 2020. The study included patients who 1) were at least 18 years old; 2) were new users of warfarin or users who resumed treatment after discontinuing warfarin for at least 12 months; 3) had been taking warfarin for 6 weeks for thromboprophylaxis of conditions such as atrial fibrillation (AF), deep venous thrombosis (DVT), pulmonary embolism (PE), mechanical heart valve (MHV), and valvular heart disease (VHD); and 3) had at least three eligible INR values, of which the interval between any two adjacent INR measurements was ≤9 weeks. The index date was set at 7 weeks after the first claim of warfarin prescription. Patients were excluded if they had an outpatient prescription filled for warfarin during the 12 months prior to the initiation of warfarin treatment. Patients were also excluded if they had <90 evaluable days or missing baseline data. The study protocol was approved by the Ethics Committee of the Second Affiliated Hospital of Chongqing Medical University (No. 2020-411).

### Anticoagulation Quality of Warfarin

We evaluated the anticoagulation quality of warfarin using TTR, which we calculated using the Rosendaal method of linear interpolation (Rosendaal et al., 1993). This method assumes a linear relationship between two consecutive INR values, assigning a specific INR value to each patient daily. After interpolation, TTR is calculated as the percentage of time during which the interpolated INR value remains within the therapeutic range. Referring to the recommendations of the prevailing antithrombotic guidelines in China (Diseases, 2018), we set the therapeutic range of INR for patients who underwent mitral valve, aortic valve, or double-valve mechanical valve replacement at 1.5–2.5, and for patients with any other indications at 2.0–3.0. A TTR of ≥60% was considered to be indicative of good anticoagulation quality, whereas a TTR of <60% was defined as poor anticoagulation quality (Hong et al., 2017). Therefore, patients were divided into two groups based on the presence of a TTR of either ≥60% or <60%.

### Outcome Measures

The primary outcome was TTR, and the secondary outcomes were clinical adverse events, including thromboembolic and bleeding events. The patients were followed up for at least three months until either a prescription was filled for a different anticoagulant or a temporary interruption in warfarin treatment occurred due to bleeding, surgery, or other invasive procedures. At least three eligible INR values were collected for each patient, and the date of the final follow-up was January 1, 2020. Thromboembolic events included stroke, transient ischemic attack (TIA), and systemic embolism (SE). Bleeding events included major and minor bleeding as defined by the criteria of the International Society on Thrombosis and Haemostasis (Schulman et al., 2010).

### Role of the Physician–Pharmacist Collaborative Anticoagulation Clinics

Some departments at our institution have specialist clinical pharmacists, and PPCCs have been established as well. The pharmacists in the PPCCs involved in this study were professional clinical pharmacists who received standardized training and obtained the corresponding certificates. In the PPCCs, physicians were responsible for patient diagnosis. Clinical pharmacists then conducted an investigation of the patients, assessing factors such as basic information; history of allergies, adverse reactions, thromboembolism, and bleeding; current therapeutic drug use; and dietary habits. Physicians and pharmacists then jointly determined the therapeutic scheme, treatment goal, course of treatment, and dosage of drugs. Finally, pharmacists provided detailed medication education to patients and informed them of the date of their next visit. After the outpatient service, pharmacists supplemented the patient’s file and followed up. In order to explore the effectiveness of PPCCs in patients with AF, we compared the anticoagulant quality of warfarin as well as clinical adverse events between patients in PPCCs and general clinics. The diagnosis of AF was based on either a standard 12-lead electrocardiogram recording or a single-lead electrocardiogram tracing of ≥30 s showing heart rhythm with no discernible repeating P-waves or irregular R-R intervals (Hindricks et al., 2020).

### Statistical Analyses

Continuous variables were presented as mean ± standard deviation and were compared using either the unpaired Student’s t-test or ANOVA tests. Categorical variables were expressed as numbers and percentages and were compared using the chi-square test or Fisher’s exact test, as appropriate. Univariate and multivariate analyses were performed to determine the potential predictors associated with poor anticoagulation quality (TTR <60%). The variables included in the univariate analysis mainly consisted of demographic characteristics, comorbidities, concomitant medication, and treatment in a PPCC setting. Due to the possible interaction with warfarin, several kinds of traditional Chinese medicine that had been reported to have influence on the efficacy of warfarin (Janetzky and Morreale, 1997; Izzat et al., 1998; Page and Lawrence, 1999; Yuan et al., 2004) were included in this study. Two criteria were considered necessary for a variable to be included in the multivariable analysis model: 1) a univariate *p* value indicative of poor anticoagulation quality ≤0.10 and 2) a plausible association with poor anticoagulation quality in patients based on data provided by the existing literature. All analyses were performed using the SPSS software, version 22.0 (SPSS Inc., Chicago, IL, United States), and *p* < 0.05 was considered to be statistically significant.

## Results

### Patient Characteristics

**FIGURE 1:**
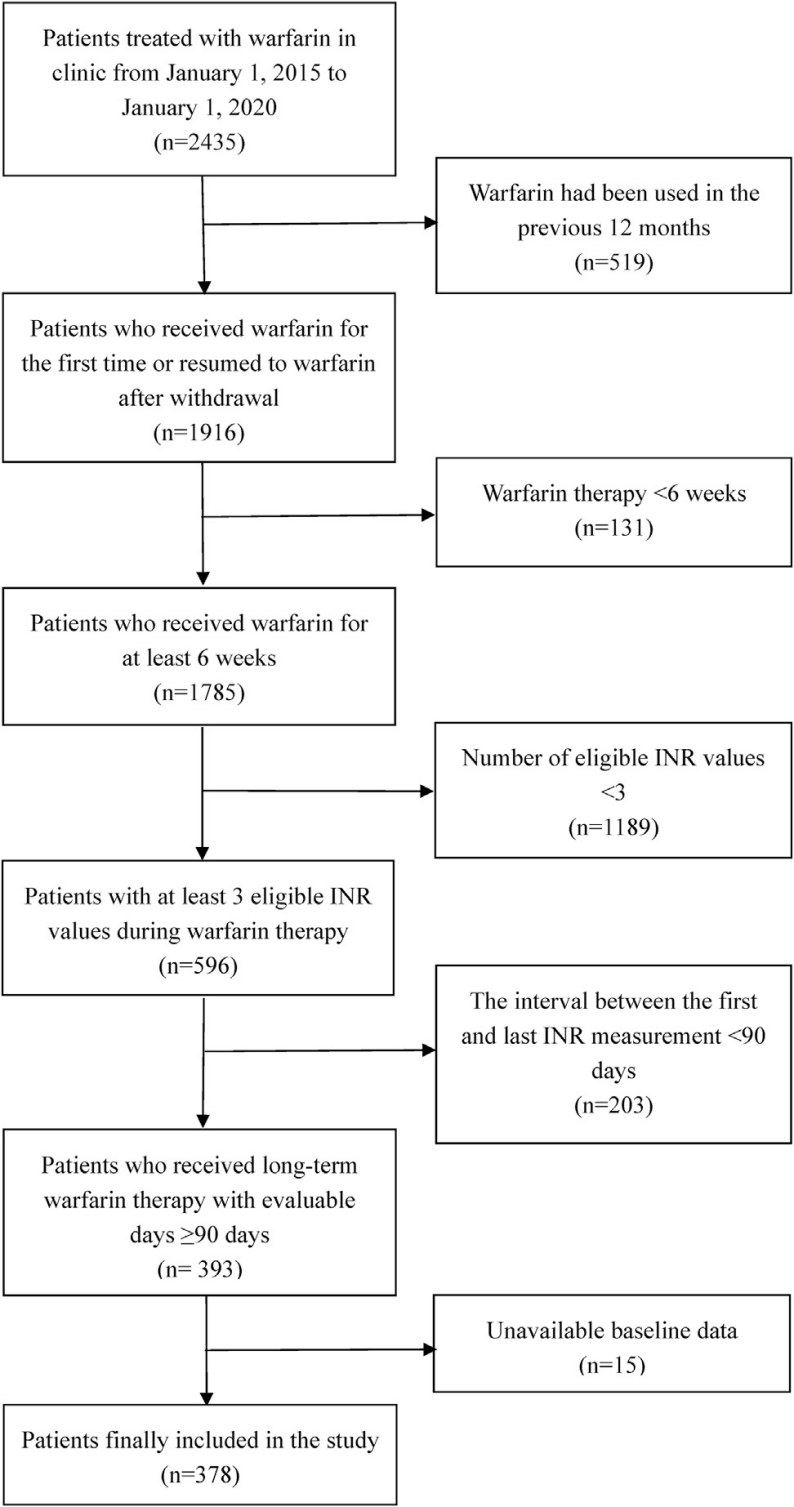
The flow diagram of selection of eligible patients. INR, international normalized ratio.

Figure 1 presents a flow diagram outlining the patient selection process. A total of 2,435 outpatients who were treated with warfarin at our clinic were reviewed between January 1, 2015 and January 1, 2020. Finally, 378 patients were included in this study. The baseline characteristics of the included patients are shown in Table 1. The mean age of the patients was 65.9 ± 12.7 years, 211 (55.8%) patients were female, and 48 (12.7%) patients regularly visited the PPCCs. Hypertension (42.9%) and coronary artery disease (27.8%) were the most common comorbidities among the included patients. The traditional Chinese medicine taken by the patients mainly consisted of astragalus (19.8%), ginseng (15.9%), and *Salvia miltiorrhiza* (16.9%). The main indications for warfarin at our institution were AF (67.7%; paroxysmal AF: 35.7%; persistent AF: 32.0%), VHD (17.2%), and DVT (13.5%).

**TABLE 1:** Demographics and characteristics of patients classified by anticoagulation quality.

Characteristics	All patients (*n* = 378)	Good anticoagulation quality (*n* = 121)	Poor anticoagulation quality (*n* = 257)	*p* Value
Age, years	65.9 ± 12.7	65.2 ± 13.8	66.3 ± 12.1	0.452
Female, n (%)	211 (55.8)	60 (49.6)	151 (58.8)	0.094
Physician-pharmacist collaborative clinic, n (%)	48 (12.7)	21 (17.4)	27 (10.5)	0.062
Comorbidities, n (%)				
Coronary artery disease	105 (27.8)	29 (24.0)	76 (29.6)	0.256
Hypertension	162 (42.9)	48 (39.7)	114 (44.4)	0.390
Diabetes	45 (11.9)	13 (10.7)	32 (12.5)	0.632
Heart failure	67 (17.7)	23 (19.0)	44 (17.1)	0.654
History of stroke	45 (11.9)	16 (13.2)	29 (11.3)	0.587
History of hemorrhage	4 (1.1)	3 (2.5)	1 (0.4)	0.099
Myocardial infarction	4 (1.1)	0 (0)	4 (1.6)	0.311
Medications, n (%)				
Antiplatelet agents	44 (11.6)	10 (8.3)	34 (13.2)	0.160
Statins	120 (31.7)	41 (33.9)	79 (30.7)	0.540
Amiodarone	17 (4.5)	5 (4.1)	12 (4.7)	0.814
Beta-blockers	157 (41.5)	52 (43.0)	105 (40.9)	0.696
ACEI or ARB	114 (30.2)	27 (22.3)	87 (33.9)	0.023
CCB	66 (17.5)	19 (15.7)	47 (18.3)	0.537
Digoxin	42 (11.1)	14 (11.6)	28 (10.9)	0.845
Traditional Chinese medicine	131 (34.7)	44 (36.4)	87 (33.9)	0.632
Astragalus	75 (19.8)	21 (17.4)	54 (21.0)	0.406
Ginseng	60 (15.9)	23 (19.0)	37 (14.4)	0.252
*Salvia miltiorrhiza*	64 (16.9)	19 (15.7)	45 (17.5)	0.662
*Angelica sinensis*	36 (9.5)	11 (9.1)	25 (9.7)	0.844
Indications for anticoagulation, n (%)				
Atrial fibrillation	256 (67.7)	85 (70.2)	171 (66.5)	0.472
Paroxysmal atrial fibrillation	135 (35.7)	46 (38.0)	89 (34.6)	0.522
Persistent atrial fibrillation	121 (32.0)	39 (32.2)	82 (31.9)	0.950
Deep venous thrombosis	51 (13.5)	14 (11.6)	37 (14.4)	0.453
Pulmonary embolism	13 (3.4)	5 (4.1)	8 (3.1)	0.763
Mechanical heart valve	29 (7.7)	11 (9.1)	18 (7.0)	0.477
Valvular heart disease	65 (17.2)	23 (19.0)	42 (16.3)	0.522

ACEI, angiotensin-converting enzyme inhibitors; ARB, angiotensin receptor blocker; CCB, calcium channel blockers; INR, international normalized ratio.

#### Anticoagulation Quality of Warfarin

**FIGURE 2:**
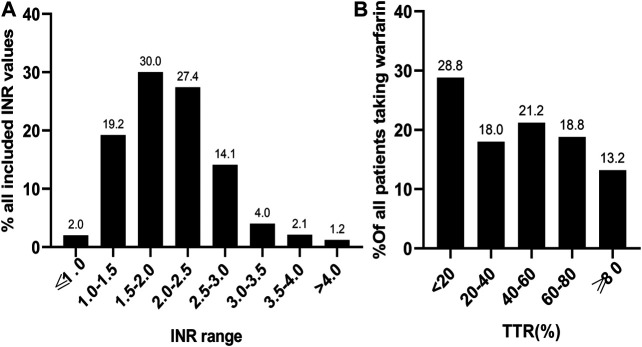
The distribution of 3,072 INR values **(A)** and TTR of 378 patients **(B)** included in the study. INR, international normalized ratio; TTR, time in therapeutic range.

**FIGURE 3:**
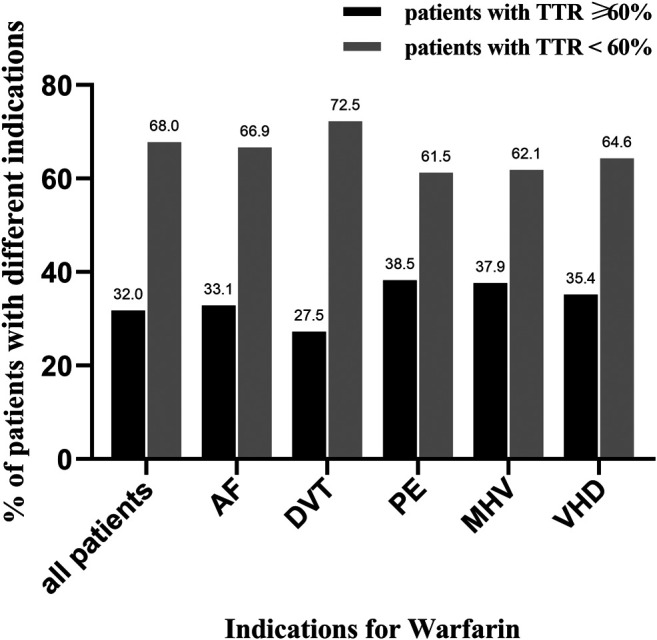
The distribution of good and poor anticoagulation quality in patients with different indications for warfarin. TTR, time in therapeutic range; AF, atrial fibrillation; DVT, deep venous thrombosis; PE, pulmonary embolism; MHV, mechanical heart valve; VHD, valvular heart disease; black column represents patients with TTR ≥ 60%, and gray column represents patients with TTR < 60%.

A total of 3,072 INR values were included in this study. The distribution of INR values is shown in Figure 2A. Most of the INR values (57.4%) were within the range of 1.5–2.5, while the INR values within the range of 2.0–3.0 only accounted for 41.5%. The mean TTR of all patients was 42.6 ± 29.8%. The distribution of TTR has been summarized in Figure 2B; patients with a TTR <20% accounted for the largest proportion (28.8%). A total of 68 (18.0%), 80 (21.2%), and 71 (18.8%) patients had TTR within the ranges of 20–40%, 40–60%, and 60–80%, respectively. Moreover, only 50 (13.2%) patients had a TTR ≥80%. Regarding the anticoagulation quality of warfarin, only 32% of patients were considered to have good anticoagulation quality, which we defined as a TTR >60%. Furthermore, consistent results were found across different indications for warfarin use (TTR ≥60%: 33.1% for AF, 27.5% for DVT, 38.5% for PE, 37.9% for MHV, and 35.4% for VHD; Figure 3).

### Potential Predictors Associated With Poor Anticoagulation Quality

A TTR of <60% was considered to be reflective of poor anticoagulation quality. The average interval of INR monitoring was represented by the average time interval between multiple consecutive sessions of INR monitoring for each patient. During the univariate regression analyses, we found that female sex, hemorrhage history, the presence of more than four comorbidities, treatment within a PPCC, ACEI/ARB use, and an average interval of INR monitoring of >30 days were statistically associated with poor anticoagulation quality (*p* < 0.1 for each variable in the univariate regression model). However, the use of traditional Chinese medicine (astragalus, ginseng, and *Salvia miltiorrhiza*) was not a potential risk factor of poor anticoagulation quality. Multivariate logistic regression analyses identified that treatment within a PPCC (OR: 0.47, 95% CI: 0.25–0.90, *p* = 0.022) was an independently protective factor related to poor anticoagulation quality; in addition, the presence of more than four comorbidities (OR: 1.98, 95% CI: 1.22–3.24, *p* = 0.006) and an average interval of INR monitoring >30 days (OR: 1.74, 95% CI: 1.10–2.76, *p* = 0.019) were independent risk factors associated with poor anticoagulation quality (Table 2).

**TABLE 2:** Predictors for poor anticoagulation quality. The bold values mean statistically significant with a P value of <0.05.

Variables	Univariate analysis			Multivariate analysis		
	OR	95% CI	*P* Value	OR	95% CI	*P* Value
Age ≥75 years	0.92	0.57–1.49	0.738			
Female	1.45	0.94–2.24	0.094	1.34	0.85–2.10	0.207
Physician–pharmacist collaborative clinic	0.56	0.30–1.04	0.062	**0.47**	**0.25–0.90**	**0.022**
History of hemorrhage	0.15	0.02–1.49	0.099	0.11	0.01–1.14	0.064
Atrial fibrillation	0.84	0.53–1.34	0.472			
Deep venous thrombosis	1.28	0.67–2.48	0.453			
Pulmonary embolism	0.74	0.24–2.33	0.612			
Mechanical heart valve	0.75	0.34–1.65	0.477			
Valvular heart disease	0.83	0.48–1.46	0.522			
≥4 comorbidities	1.97	1.24–3.13	0.004	**1.98**	**1.22–3.24**	**0.006**
Antiplatelet agents	1.69	0.81–3.55	0.168			
Statins	0.87	0.55–1.37	0.540			
Amiodarone	1.14	0.39–3.30	0.817			
Beta blockers	0.92	0.59–1.42	0.696			
ACEI or ARB	1.78	1.08–2.94	0.023	1.56	0.92–2.63	0.096
CCB	1.20	0.67–2.15	0.537			
Digoxin	0.93	0.47–1.85	0.845			
Traditional Chinese medicine	0.90	0.57–1.41	0.632			
Average interval of INR monitoring >30 days	1.96	1.25–3.06	0.009	**1.74**	**1.10–2.76**	**0.019**

ACEI, angiotensin converting enzyme inhibitors; ARB, angiotensin receptor blocker; CCB, calcium channel blockers; INR, international normalized ratio; OR, odds ratio, CI, confidence interval.

### Clinical Adverse Events During Follow-Up

During a mean follow-up period of 192 ± 92 days, 33 patients (8.5%) experienced thromboembolic or bleeding events during their treatment with warfarin (Table 3). There were no significant differences in the incidences of thromboembolic events (5.0% vs. 5.1%, *p* = 0.967) and bleeding events (1.7% vs. 4.7%, *p* = 0.241) between patients with good anticoagulation quality and those with poor anticoagulation quality. Severe thromboembolic events consisted primarily of ischemic stroke (12 patients) and myocardial infarction (two patients). Among the 14 patients who experienced bleeding events, five patients experienced major bleeding (three cases of hematuria and 4 cases of gastrointestinal bleeding), and the rest suffered only minor bleeding, such as epistaxis and subconjunctival hemorrhage.

**TABLE 3:** Comparison of clinical outcomes of good anticoagulation quality vs. poor anticoagulation quality.

Outcomes	Good anticoagulation quality (*n* = 121)	Poor anticoagulation quality (*n* = 257)	*p* Value
Thromboembolic events, n (%)	6 (5.0)	13 (5.1)	0.967
Stroke	4 (3.3)	8 (3.1)	1.000
Myocardial infarction	1 (0.8)	1 (0.4)	0.538
Peripheral venous thrombosis	1 (0.8)	3 (1.2)	1.000
Peripheral artery thrombosis	0 (0.0)	1 (0.4)	1.000
Bleeding events, n (%)	2 (1.7)	12 (4.7)	0.241
Epistaxis	0 (0.0)	2 (0.8)	1.000
Hemoptysis	0 (0.0)	1 (0.4)	1.000
Hematuria	1 (0.8)	2 (0.8)	1.000
Gastrointestinal bleeding	0 (0.0)	4 (1.6)	0.311
Subcutaneous bleeding	1 (0.8)	2 (0.8)	1.000
Subconjunctival bleeding	0 (0.0)	1 (0.4)	1.000

### Comparison of Anticoagulation Quality Between Physician–Pharmacist Collaborative Clinics and General Clinics in Atrial Fibrillation

Finally, in order to compare the PPCCs and general clinics, we included 48 patients with AF from the PPCCs and 208 patients with AF undergoing treatment in general clinics (Table 4). The baseline characteristics between the two groups were relatively similar, except for age and beta-blocker use (*p* = 0.03 and *p* = 0.024, respectively). The mean TTR was significantly higher in patients being treated at the PPCCs than in the general outpatients (48.4% ± 25.7% vs. 38.0% ± 27.6%, *p* = 0.014). As the sample size was limited, the number of patients with good anticoagulation quality was higher in the PPCC group than in the general clinics group (43.75% vs. 30.8%, *p* = 0.085). Since the presence of more than four comorbidities and an average interval of INR monitoring >30 days were identified as independent risk factors for poor anticoagulation quality, we performed further analyses based on the above two factors. For patients with more than four comorbidities, no significant differences were observed between patients from PPCCs and those from general clinics with respect to mean TTR (48.4% ± 25.7% vs. 38.0% ± 27.6%, *p* = 0.097) and the proportion of good anticoagulation quality (34.8% vs. 23.6%, *p* = 0.256). Regarding patients with an average interval of INR monitoring of >30 days, both the mean TTR (54.3% ± 26.8% vs. 35.5% ± 28.1%, *p* = 0.005) and proportion of good anticoagulation quality (42.9% vs. 20.9%, *p* = 0.032) were higher in patients from PPCCs than in the general outpatients. The incidences of thromboembolic and bleeding events did not differ between the two groups (*p* > 0.05 for each outcome).

**TABLE 4:** Comparison between patients with AF in PPCCs and patients with AF in general clinics. The bold values mean statistically significant with a P value of <0.05.

Characteristics	Patients with AF in physician-pharmacist collaborative clinics (*n* = 48)	Patients with AF in general clinics (*n* = 208)	*p* Value
Age, years	67.0 ± 10.4	70.4 ± 9.5	**0.030**
Female, n (%)	28 (58.3)	116 (55.8)	0.747
Paroxysmal atrial fibrillation, n (%)	26 (54.2)	109 (52.4)	0.825
Coronary artery disease, n (%)	17 (35.4)	80 (38.5)	0.695
Hypertension, n (%)	30 (62.5)	116 (55.8)	0.396
Diabetes, n (%)	10 (20.8)	29 (13.9)	0.231
Heart failure, n (%)	9 (18.8)	47 (22.6)	0.561
History of stroke, n (%)	5 (10.4)	31 (14.9)	0.420
History of bleeding, n (%)	0 (0)	3 (1.4)	1.000
Myocardial infarction, n (%)	0 (0)	4 (1.9)	1.000
Antiplatelet agents, n (%)	3 (6.2)	29 (13.9)	0.224
Statins, n (%)	21 (43.8)	87 (41.8)	0.808
Amiodarone, n (%)	3 (6.2)	13 (6.3)	1.000
Beta-blockers, n (%)	32 (66.7)	101 (48.6)	**0.024**
ACEI or ARB, n (%)	19 (39.6)	84 (40.4)	0.919
CCB, n (%)	14 (29.2)	46 (22.1)	0.299
Digoxin, n (%)	7 (14.6)	21 (10.1)	0.369
Traditional Chinese medicine, n (%)	10 (20.8)	68 (32.7)	0.108
Outcome measures			
Mean TTR	53.2% ± 29.6%	41.6% ± 29.2%	**0.014**
Good anticoagulation quality, n (%)	21 (43.75)	64 (30.8)	0.085
≥ 4 comorbidities, n (%)	23 (47.9)	106 (51.0)	0.704
Mean TTR	48.4% ± 25.7%	38.0% ± 27.6%	0.097
Good anticoagulation quality, n	8	25	0.256
Average interval of INR monitoring > 30 days, n (%)	21 (43.8)	110 (52.9)	0.254
Mean TTR	54.3% ± 26.8%	35.5% ± 28.1%	**0.005**
good anticoagulation quality, n	9	23	**0.032**
Thromboembolic events, n (%)	3 (6.2)	12 (5.8)	1.000
Hemorrhagic events, n (%)	3 (6.2)	9 (4.3)	0.703

AF, atrial fibrillation; ACEI, angiotensin-converting enzyme inhibitors; ARB, angiotensin receptor blocker; CCB, calcium channel blockers; INR, international normalized ratio; TTR, time in therapeutic range.

## Discussion

NOACs are currently recommended as the optimal anticoagulation treatment, with similar or lower risks of stroke, systemic embolism, major bleeding, and death compared with warfarin (Miller et al., 2012; Coleman et al., 2017). However, the use of NOACs in China is limited by many objective factors, such as high prices, the limits imposed by medical insurance indications, the relative complexity of dose adjustment based on specific factors (e.g., renal function and age), and concomitant medications. Currently, warfarin is widely used as an oral anticoagulant; hence, it is necessary to investigate and analyze the anticoagulation quality of warfarin. In the present study, we retrospectively evaluated the anticoagulation quality of warfarin in 378 patients from our outpatient department. We found that the mean TTR of the included patients was 42.6. ± 29.8%, and only 121 (32%) patients achieved good anticoagulation quality (TTR≥ 60%). In addition, both the presence of more than four comorbidities and an average interval of INR monitoring of >30 days were identified as independent risk factors for poor anticoagulation quality. On the contrary, treatment at one of the PPCCs was an independent protective factor for poor anticoagulation quality. Finally, we confirmed that the PPCC model significantly improved the anticoagulation quality of warfarin compared to general clinics.

Data from the ROCKET AF trial found that high-quality anticoagulant therapy was the key to ensuring the efficacy and safety of warfarin administration (Singer et al., 2013). To date, several retrospective studies have been conducted to evaluate the anticoagulation quality of warfarin in different countries using the same TTR calculation as that of the present report; moreover, these studies included patients who were undergoing continuous warfarin treatment for the first time. In one study, a mean TTR of 76.2% was reported, and a TTR of >70% was consistently observed across all age-groups within a cohort of 18,391 patients from 67 different centers in Sweden (Wieloch et al., 2011). In another report on 3,692 AF patients in Australia, 97% of the cohort had TTRs exceeding 60%, and a mean TTR of 81% was observed (Bernaitis et al., 2016). The Swedes and Australians seemed to have superior anticoagulation quality as a result of warfarin administration. By contrast, an American prospective observational study involving 5,210 patients on warfarin at 155 sites reported a markedly lower mean TTR of 65% (Pokorney et al., 2015). Considering that prior studies have reported that the Asian population generally showed abnormal sensitivity to warfarin and had a higher risk of major bleeding than Western populations, the administration of warfarin in Asian patients has proven to be a challenge (Shen et al., 2007; Singer et al., 2013). Compared to Japanese patients (mean TTR of 64%) (Okumura et al., 2011), the anticoagulation quality of warfarin was worse in Koreans, with a mean TTR of 49.1%; moreover, only 31% of patients showed good anticoagulation control (TTR> 60%) (Hong et al., 2017). In comparison to the aforementioned studies, we found that the mean TTR at our institution was very low, which was similar to that of the Korean patients. Therefore, it is urgent that our institution carry out anticoagulant management services in order to improve anticoagulation quality.

Clinical factors have been proven to be associated with anticoagulation quality. Renal insufficiency, advanced heart failure, frailty, history of valve surgery, and high risk of stroke might contribute to poor anticoagulation control in Americans (Pokorney et al., 2015). Different factors, such as female sex, age, the presence of more than two comorbidities, and smoker status, were found to be indicators of poor INR control in patients from the United Kingdom (Apostolakis et al., 2013). With regard to African patients, hypertension (the most common comorbidity) was an important factor related to anticoagulation quality (Mwita et al., 2018). In Asian patients, both patient age and National Institutes of Health Stroke Scale scores were found to be associated with poor anticoagulation quality in Japan (Okumura et al., 2011) and Korea (Hong et al., 2017). In the present study, the findings of the multivariate logistic regression model indicated that both the presence of more than four comorbidities and an average interval of INR monitoring >30 days were potential risk predictors associated with poor anticoagulation quality. Regarding the comorbidities, several studies have demonstrated that the coexistence of multiple diseases could significantly reduce the quality of coagulation control in patients taking warfarin. This can be explained by the increased yearly number of hospitalizations required due to the comorbidities, which, in turn, gave rise to poor anticoagulation quality in warfarin users (Davis et al., 2005; Apostolakis et al., 2013). In this study, most of the included patients had additional complications, such as coronary heart disease, heart failure, hypertension, and diabetes, resulting in repeated hospitalizations which would inevitably impact the efficacy of warfarin. Thus, the incidence of poor anticoagulation quality could be increased by 98% in patients with more than four comorbidities. Accordingly, it is more difficult for patients with multiple comorbidities to reach the effective anticoagulant range for warfarin. In this study, the use of the time interval between INR monitoring sessions was considered a suitable measure, as it ensured relatively frequent monitoring and better TTR control. When monitored both monthly and weekly, we expect that the number of patients who would remain in the therapeutic range would be 50–60% and 77–85%, respectively. When referring to existing research, it appears that the compliance rate of patients could potentially reach 92% every 3 days, given that sufficient monitoring is performed (Khan et al., 2004). The American Heart Association recommended that the interval of INR monitoring should not exceed four weeks for patients undergoing long-term warfarin therapy (Dolan et al., 2008). Our study also found that an interval of INR monitoring of more than 30 days increased the risk of poor anticoagulation quality. The interval of INR monitoring may also reflect the compliance of patients, which, in turn, could be improved by enhancing patients’ knowledge of warfarin.

In our study, multivariate regression analysis showed a positive effect of PPCCs on the anticoagulation quality of warfarin, which was consistent with the results of the comparison of anticoagulation quality between patients from PPCCs and those from general clinics. After eliminating the influence of comorbidities and the length of the interval of INR, pharmacists were still able to improve TTR control levels in patients with AF, which suggests that the participation of clinical pharmacists in anticoagulation management could bring about certain benefits. Anticoagulation clinics led by pharmacists have been reported previously, and some success has been achieved in certain countries, such as the United States, New Zealand, and South Korea (Choe et al., 2002; Harrison et al., 2015; Rose et al., 2017). Previous studies found that anticoagulation clinics managed by pharmacists were able to increase the compliance rate of patients undergoing anticoagulant therapy from 48–50% to 58–76% (*p* < 0.001) (Garwood et al., 2008; Gupta et al., 2015). Notably, anticoagulation management is mainly the domain of physicians, and the possibility of clinical pharmacists to participate in this treatment is in its early stages in China. A limited number of cooperative anticoagulation clinics have been established in tertiary hospitals in China, such as those of the Nanjing Drum Tower Hospital, Fujian Medical University Union Hospital, Shanghai Renji Hospital, Fuwai Hospital of the Chinese Academy of Medical Sciences, and Peking Union Medical College Hospital. In our hospital, few clinical departments have implemented the PPCC model. Overall, clinical pharmacists in our institution could play an important role in improving the quality of warfarin therapy by assisting patients with multiple comorbidities in drug management as well as by strengthening medication education. The results of this study could provide the basis for the establishment of professional anticoagulation clinics in our hospital in the future.

There were some limitations to this study. First, this was a retrospective, observational, single-center study with a limited number of samples and a relatively short follow-up period. Thus, causal inference relating to clinical adverse outcomes was limited. Second, we did not collect information pertaining to dose adjustment or patient genotypes (VKORC1 and CYP2C9), which limited the possibility of further analysis regarding the influence of coagulation on the above variables. Third, the variable number of INR values collected retrospectively for each patient affected the results of the subsequent TTR calculations. Fourth, due to the retrospective nature of the data collection, the duration of treatment with warfarin might have been inconsistent, ranging from 6 to 12 months. Finally, methods that adjust for confounding variables, such as propensity-matched comparison, might not be possible due to the limited sample size. To some degree, this could be a potential source of bias.

## Conclusion

In the real-world setting of our institution, the anticoagulation quality of warfarin was relatively low. Certain factors, such as the presence of more than four comorbidities and an average interval of INR monitoring of >30 days, were independent indicators of poor anticoagulation quality. The PPCC model positively impacted anticoagulation quality and is, therefore, a promising direction for future treatment.

## Data Availability Statement

The raw data supporting the conclusions of this article will be made available by the authors, without undue reservation.

## Ethics Statement

The studies involving human participants were reviewed and approved by the Second Affiliated Hospital of Chongqing Medical University. The Ethics Committee waived the requirement of written informed consent for participation. Written informed consent was not obtained from the individual(s) for the publication of any potentially identifiable images or data included in this article.

## Author Contributions

Z-CG is the guarantor of the entire manuscript. Z-CG, SQ, and NW contributed to the study conception and design, critical revision of the manuscript for important intellectual content, and final approval of the version to be published. CZ and YQ contributed to the data acquisition, analysis, and interpretation.

## Funding

This study was supported by Social Undertakings and Livelihood Security Science and Technology Innovation Project of Chongqing Science and Technology Commission (cstc2017shmsA130041), Research Funds of Shanghai Health and Family Planning commission (20184Y0022), Cultivation fund of clinical research of Renji Hospital (PY2018-III-06), and Clinical Pharmacy Innovation Research Institute of Shanghai Jiao Tong University School of Medicine (CXYJY2019ZD001, CXYJY2019QN004).

## Conflict of Interest

The authors declare that the research was conducted in the absence of any commercial or financial relationships that could be construed as a potential conflict of interest.
